# Differences in Sweet Taste Perception and Its Association with the *Streptococcus mutans* Cariogenic Profile in Preschool Children with Caries

**DOI:** 10.3390/nu12092592

**Published:** 2020-08-26

**Authors:** Anna Jurczak, Małgorzata Jamka-Kasprzyk, Zuzanna Bębenek, Małgorzata Staszczyk, Paweł Jagielski, Dorota Kościelniak, Iwona Gregorczyk-Maga, Iwona Kołodziej, Magdalena Kępisty, Magdalena Kukurba-Setkowicz, Amira Bryll, Wirginia Krzyściak

**Affiliations:** 1 Department of Pediatric Dentistry, Institute of Dentistry, Jagiellonian University Medical College, Montelupich 4, 31-155 Cracow, Poland; anna.jurczak@uj.edu.pl (A.J.); malgorzata.jamka-kasprzyk@uj.edu.pl (M.J.-K.); malgorzata.staszczyk@uj.edu.pl (M.S.); dorota.koscielniak@uj.edu.pl (D.K.); iwona.gregorczyk-maga@uj.edu.pl (I.G.-M.); iwona.kolodziej@uj.edu.pl (I.K.); magdalena.kepisty@uj.edu.pl (M.K.); magdalena.kukurba-setkowicz@uj.edu.pl (M.K.-S.); 2 Department of Mycology, Collegium Medicum, Jagiellonian University, Czysta St 18, 31-121 Cracow, Poland; zuzanna.bebenek@uj.edu.pl; 3 Department of Nutrition and Drug Research, Faculty of Health Science, Collegium Medicum, Jagiellonian University, Grzegórzecka St 20, 31-531 Cracow, Poland; paweljan.jagielski@uj.edu.pl; 4 Department of Radiology, Jagiellonian University Medical College, Kopernika 19, 31-501 Cracow, Poland; bryllamira@gmail.com; 5 Department of Medical Diagnostics, Faculty of Pharmacy, Jagiellonian University Medical College, Medyczna 9, 30-688 Cracow, Poland

**Keywords:** Taste preferences, eating habits, microbial dysbiosis, early childhood caries (ECC)

## Abstract

The aim of the study was to verify the hypothesis about differences in sweet taste perception in the group of preschool children with and without caries, and to determine its relationship with cariogenic microbiota and the frequency of sweets consumption in children. The study group included of 63 children aged 2–6 years: 32 with caries and 31 without caries. The study consisted of collecting questionnaire data and assessment of dental status using the decayed, missing, filled in primary teeth index (dmft) and the International Caries Detection and Assessment System (ICDAS II). The evaluation of sweet taste perception was carried out using a specific method that simultaneously assessed the level of taste preferences and the sensitivity threshold for a given taste. The microbiological analysis consisted of the assessment of the quantitative and qualitative compositions of the oral microbiota of the examined children. The sweet taste perception of children with caries was characterized by a lower susceptibility to sucrose (the preferred sucrose solution concentration was >4 g/L) compared to children without caries (in the range ≤ 4 g/L, *p* = 0.0015, chi-square test). A similar relationship was also observed for frequent snacking between meals (*p* = 0.0038, chi-square test). The analysis of studied variables showed the existence of a strong positive correlation between the perception of sweet taste and the occurrence and intensity of the cariogenic process (*p* = 0.007 for dmft; and *p* = 0.012 for ICDAS II), as well as the frequency of consuming sweets (*p* ≤ 0.001 for frequent and repeated consumption of sweets during the day, Spearman test) in children with caries. Additionally, children with an elevated sucrose taste threshold were more than 10-times more likely to develop *S. mutans* presence (OR = 10.21; 95% CI 3.11–33.44). The results of this study suggest the future use of taste preferences in children as a diagnostic tool for the early detection of increased susceptibility to caries through microbial dysbiosis towards specific species of microorganisms.

## 1. Introduction

Oral health is an integral part of overall health and wellness. The growing problem of early childhood caries (ECC) has led researchers to focus on the complex, multifactorial nature of the disease. Its key causes include poor eating habits, in particular frequent consumption of sugars, which are metabolized by oral microorganisms, e.g., *S. mutans*, that take part in the formation of cariogenic biofilms [1]. The development of the disease involving the perception of a sweet taste, which affects the frequency of sugar consumption, and thus a greater predisposition to caries in early childhood, is also of importance [2].

The first months of a baby’s life are key to learning taste. Early experiences with different tastes shape the acquired preferences which are important for the later acceptance of food, especially healthy food. Available studies show that proper nutrition of mothers during pregnancy, lactation, and in the first years of the child’s life not only ensures adequate weight gain and optimal nutritional status, but also influences the long-term effects of the so-called health ‘programming’ of the metabolism, conditioning proper future health, also of the oral cavity [3,4].

Still in the womb, a baby gains its first taste experiences in this prenatal period, learning the flavors of food consumed by the mother, thus influencing later preferences in this regard. In this way, it also experiences cultural and environmental taste patterns long before the period when it begins direct contact proper with food [5].

Babies naturally prefer sweet tastes, which allows them to accept the mother’s natural, slightly sweet food. This results from the body’s physiological response to the consumption of sweet-tasting foods, which is the production of endorphins and serotonin, i.e., the so-called happiness hormones improving mood and memory in cases of good glucose tolerance [6]. This explains why children are at high risk of overconsuming sugar [7]. Too much sugar in a child’s diet, sugar added to foods and drinks or found naturally in unsweetened fruit juices, honey or syrups, may, however, impair the absorption of important elements, e.g., iron and zinc, as well as suppress the appetite, hindering proper growth and development due to a lack of nutrients.

A woman’s nutrition during pregnancy and lactation is therefore important in learning about new tastes and shaping the child’s preferences in this respect [8,9]. Research indicates that breastfed infants accept the introduction of complementary foods more easily. Mother’s milk is rich in flavors and aromas (depending on the diet of the nursing woman), which has a significant impact on the future preferences of the child [10,11].

Infants aged 4–7 months are the most open to new flavors; at that time new products are accepted by children much faster compared to later ages [12,13]. For this reason, the timing of diet expansion seems to be important in shaping eating habits [14]. The World Health Organization (WHO) recommends giving infants unsweetened complementary foods, not only because of the long-term impact on the health of the child, but also for the formation of appropriate habits and nutritional preferences that appear in later years [15]. One of the most common nutritional mistakes made when modifying the diet is sweetening meals and drinks to adjust the child’s taste to the tastes of adults, while children at this age willingly accept a neutral taste [16].

Improper nutritional behaviors in children change throughout their individual lives, but also have wide-ranging consequences, from behavioral problems related to anorexia, overeating, or nutritional neophobia, to significant health problems such as increased risk of obesity, endocrine problems, anemia, idiopathic shortness of growth, cardiovascular diseases, type 2 diabetes mellitus (T2DM), and other metabolic disorders and ECC [4,17,18,19,20,21,22,23]. A strong correlation has been proven between excessive sugar supply with the risk of caries [24], which is considered a social disease of a progressive nature, affecting people of all ages all over the world and which is considered the most common infectious disease among children [25]. An important role in its formation is played by social factors, such as diet, and interactions between the consumed food, the host, and carious microorganisms, such as *Streptococcus mutans*, which produce acids from fermentable sugars on the tooth enamel surface [26,27].

*Streptococcus mutans,* as a recognized etiological factor of dental caries, participate in initiating the formation of the dental biofilm structure. Biofilms are multicellular creatures composed of many kinds or species of microorganisms arranged in layers [28]. They are permanently attached to each other and to the substrate surrounded by a layer of extracellular polysaccharides and proteins. The microorganisms suspended in the form of a biofilm show different characteristics from their planktonic forms, such as increased resistance of biofilm to chemotherapeutic agents, compared to free-living forms [29]. Polysaccharides present in the matrix increase the adherence of microorganisms and ensure the integrity of the biofilm. They can serve as a backup source of energy in times of hunger and protect the microorganisms concentrated in the biofilm against hostile influences. They also affect the diffusion of substances to and from the biofilm, as well as facilitate concentration of metal ions and nutrients in the biofilm environment [30].

Biofilm development is a dynamic process that has several stages that intertwine with each other. It depends on the conditions in the oral cavity and changes over time [31]. Bacterial plaque is a natural phenomenon and is, under certain conditions, beneficial to human health [32]; however, due to changes in the local environment of the oral cavity, mainly under the influence of carbohydrates supplied from the diet, it can lead to dysbiosis. This is a disease process that initially manifests itself in a change in the species composition of commensal microbiota in favor of acid-forming microorganisms leading to the development of cavitation changes in tooth enamel [33,34]. Although *S. mutans* and, to a lesser extent, *S. sobrinus* are associated with the initiation of carious tooth lesions, the latest omics developments highlight the contribution of a wider range of acidophilic species, including other streptococci, *Actinomyces* spp. and Bifidobacteria such as *Scardovia wiggsiae*, in the development of dental caries [35,36,37].

Biofilms are a unique and complex spatial structure composed of bacteria, fungi, and viruses. Studies on the oral microflora show that biofilms created in various locations (tongue, cheeks, tooth surfaces) are characterized by both phenotypic and genotypic variability of the local microbiota [36]. Information about the spatial distribution and interactions between the species found in different locations of oral biofilms is still incomplete [38]. Modern technologies of spatial imaging and omics reveal the unique spatial properties of the structures forming biofilms on the teeth of children with caries. These studies are a breakthrough in the understanding of the variability of the composition of the oral microflora depending on the surrounding environment related to the period of an individual’s life. The latter, in turn, depends on the patterns of structures forming biofilms from mixed bacterial and fungal clusters with strong aggregating properties to spatial multilayer oral biofilm architectures [33]. This information is of extreme interest in situations where the age of human microbiome determines the development of the disease process and related therapeutic strategies.

The oral cavity is one of the most colonized microbiomes of the human organism in terms of species diversity [38]. The microbiome of the human oral cavity consists of a difficult to determine number of microorganisms representing distinct ecological niches limited anatomically, e.g., microbiota of the surface of the tongue, cheek, teeth, palate, etc. The factors determining the variable composition of oral microbes, apart from the anatomical structure, are: saliva quality, eating habits, and the taste preferences that are the subject of this study [39].

During the individual’s life, the environment of the oral cavity is constantly changing depending on age, the appearance of the first teeth, their carious cavities, dentures, fillings, tooth absence, as well as the changes related to eating habits, weakened saliva flow, or chronic pharmacotherapy [40]. Local environmental conditions, such as temperature, salinity, availability of oxygen, nutrients, and changing pH conditions, may affect the ecosystem and contribute to a change in the species composition of biofilms [41]. The oral cavity microbiota is shaped in stages during an individual’s life, starting from birth through contact between the surface of the newborn’s skin and oral mucosa with the mother’s vaginal microbiota. In the case of delivery by caesarean section, microflora is transferred from the mother’s skin to the surface of the newborn’s skin and mucosa [42].

Immediately after birth, bacterial species that cover most of the body surface of the newborn (mouth, nasopharynx, skin, and intestine) are very similar to each other [43]. Children born naturally have an oral microbiome similar to that of the mother’s vagina, while children born by caesarean section have a bacterial flora similar to that present on the mother’s skin [44].

During the perinatal period, the oral cavity is massively exposed to microorganisms from the outside world. This is the time when the process of permanent oral colonization begins. During the first 24 h of a newborn’s life, the so-called pioneering microorganisms that determine the composition of the oral microbiome are being set. At this stage, the most common colonizers of the oral cavity are Gram-positive cocci, including the *Streptococcus* (including: *S. sanguinis, S. mitis, S. oralis,* and *S. salivarius*) and Staphylococcus genera [45].

Pioneering microorganisms initiate changes in the environment by producing and secreting products of their metabolism, which often entails the growth of other species. *Streptococcus salivarius* present in the oral cavity of newborns promotes the growth of other species of bacteria, including *Actinomyces* spp. that may adhere to each other [46], contributing to the formation of a more stable structure of the oral microenvironment.

During the first two months of a child’s life, bacteria only colonize the surfaces of the mucous membranes. Along with the eruption of the milk dentition, new species of microorganisms appear, which determine changes in the oral cavity microbiome [28]. As the child grows, the oral microbiome evolves. At around five months of age, the oral microflora has a marked resemblance to that of the mother’s mouth due to environmental conditions that occur in the first months of life, in particular through feeding, contact with other adults and children, eating habits, and sweet taste preferences [47]. The microflora of the oral cavity of the newborn then mainly consists of bacteria, including six clusters: Firmicutes, Proteobacteria, Actinobacteria, Bacteroidetes, Fusobacteria, and Spirochaetes. The most numerous are: Streptococcus, Haemophilus, Neisseria, and Veillonella [48]. Interestingly, some of these microorganisms, such as *S. mitis* and *S. oralis*, produce anti-immunoglobulin (IgA) proteases that specifically degrade salivary IgA secretion. Thanks to this function, microorganisms are able to survive in an environment rich in IgA, which is excreted in breast milk [49]. Although infants have fewer microorganisms in their mouths during this period than their parents, their species composition is richer [48].

During the eruption of the dentition, a new ecological niche is formed. It is likely that colonization of cariogenic *Streptococcus* spp. strains such as *S. mutans* (which are the subject of this study) begins at the stage when hard surfaces appear. This moment was described by Caufield et al. as the so-called “infectivity window” [50]. Recent studies, however, refute the above-mentioned view showing the presence of this species in toothless children, which suggests that soft tissues can be a reservoir for pathogenic microorganisms [43]. This fact emphasizes the importance of eating habits and the preference to sweet taste at such an early stage in a child’s life: before the eruption of the dentition.

The oral microbiome appears to be complete around three years of age, but the maturation process continues into adulthood. The bacterial flora of a child’s mouth changes during the development of milk, mixed, or permanent dentition. The oral microflora in children with milk dentition shows a higher frequency of bacteria belonging to the families Pseudomonaceae (Pseudomonas genus), Moraxellaceae (Acinetobacter, Moraxella, and Enhydrobacter genera), Enterobacteriaceae, and Pasteurellaceae (Aggregatibacter genus) compared to other groups. When the milk dentition is replaced permanently, the population of bacteria belonging to the Veillonellaceae family and the Prevotella genus increases, while the Carnobacteriaceae family decreases [51].

The population of oral microorganisms in children aged 3–12 consists of bacterial species inhabiting various ecological niches, e.g., the surface of the cheeks, gums, and tongue. Moreover, their composition in these locations is very diverse [52]. This is evidenced by observations [37] which have shown that 10% of examined children and adolescents with caries have no *S. mutans*. Moreover, the participation of other bacterial species in the development and progression of caries, e.g., Lactobacillus, Veillonella, Bifidobacterium, Propionibacterium, acid-forming species independent of Streptococcus mutans (*S. gordonii*, *S. oralis*, *S. mitis*, and *S. anginosus*), Actinomyces, and Atopobium, demonstrates the effect of mixed bacterial flora associated with different habitants on the development of dental caries [53]. The species of Streptococcus and Actinomyces are the best studied etiological factors of enamel damage. In the absence of *S. mutans* and Lactobacillus, initial demineralization of the enamel may also occur due to *S. sanguinis, S. mitis*, and *S. oralis*, which are early colonizers [54].

On the other hand, when dentin lesions appear, the proportion of *S. mutans* accounts for about 30% of the total microflora, which indicates that it is associated with the advanced stage of tooth damage. However, *S. mutans* is less common in caries progression, with Lactobacillus, Bifidobacterium, and Prevotella species dominating instead [55]. Studies evaluating the composition of the microflora associated with early childhood caries show the main share of bacteria to be from the genera are Streptococcus, Veillonella, Actinomyces, Propionibacterium, Granulicatella, Leptotrichia, Thiomonas, Bifidobacterium, and Atopobium, constituting the physiological flora. The virulence of a population of cariogenic bacteria correlates with the adopted phenotype for a given environment related to the potential of acid-forming bacteria.

Under physiological conditions, the oral cavity of children has a higher percentage of bacteria belonging to the divisions Firmicutes (Streptococcus, Veillonella, Lactobacillus, and Granulicatella genera) and Actinobacteria (Rothia and Actinomyces genera), and a lower percentage of bacteria from the divisions Bacteroidetes (Bacteroidales and Prevotella genera), Fusobacteria (Fusobacterium genus), Spirochaetes, and the transmembrane segment 7 (TM7) strain compared to adults [51]. Interestingly, as the child grows, dysbiosis develops towards cariogenic bacteria. This change concerns the reduction of the population of aerobic or facultative Gram-positive cocci in favor of Gram-negative anaerobic bacteria [56].

Puberty is a time of major hormonal changes that accompany the enrichment of the oral environment with a variety of nutrients. This leads to the growth of some groups of microorganisms, including Gram-negative anaerobic bacteria and spirochetes [57]. This alteration of the oral flora may be associated with an increased incidence and severity of gingivitis during puberty [58].

It takes several years for the permanent teeth to erupt and for a complete oral microbiome to develop. Despite the variable composition of the oral microflora during an individual’s life, it should be borne in mind that current research on the oral microbiome does not allow a full assessment of the presence and viability of the cells. The mere determination of the presence of a given species does not mean that it is a permanent inhabitant of the oral microbiota and that its presence determines the appearance of the disease.

Predisposition to the occurrence of caries, although genetically determined, may contribute to its development due to improper eating behavior related to e.g., an excessive supply of simple sugars, shaped as early as in the prenatal period. The consequences are significant as they may result in the activation of toll-like receptor 4/nuclear factor kappa-light-chain-enhancer of activated B-cells (TLR4/NF-κB) signaling, leading to inhibition of neurogenesis in the developing central nervous system [59,60].

Thus, the preferences for a sweet taste, on the one hand, are the result of genetic predispositions, but on the other hand, early experiences, both in prenatal life and infancy, may modify them [61,62]. Moreover, a number of socioeconomic factors, host immunity, personal hygiene, availability of dental care, and fluoride prophylaxis determine different levels of effectiveness of measures to prevent the formation of cariogenic biofilms that promote caries development [63]. A diet rich in carbohydrates, especially sucrose, seems to be a key factor in increasing the virulence of the formed plaque [64]. Better understanding of the complicated process of caries may contribute to the inclusion of new methods of preventing this disease, as well as the improvement of existing procedures. The use of methods of assessing taste preferences in young children, which determine the predisposition to the formation of cariogenic plaque biofilms, seems to be particularly promising.

The presented research focused on the assessment of the influence of sucrose as a ‘default’ food sugar. Natural sweeteners from the group of polyhydric alcohols, especially xylitol [65,66,67,68], have a significant impact on the inhibition of caries development. Artificial sweeteners and their connection with the intensification or inhibition of the development of caries have been the subject of many studies which have shown a lower cariogenic potential of these substances compared to sucrose [69]. However, no studies have investigated the influence of artificial sweeteners on the perception of sweet taste and the oral cavity microbiota, in both adults and children, and this seems to be an interesting research topic for the future.

The aim of the study was to verify the hypothesis about the differences in the perception of sweet taste (based on sucrose as a traditional food sugar) in children with and without caries, and to determine its relationship with the cariogenic microflora and the frequency of children’s consumption of sweets.

## 2. Materials and Methods

### 2.1. Study Group and Study Design

The study group consisted of 63 children aged 2–6 years, 47.6% of whom were boys and 52.4% girls. There were 32 children with caries and 31 children without caries (control group). Clinical examinations were conducted by a specialist in pediatric dentistry with experience in oral health research at the Department of Pediatric Dentistry, University Dental Clinic in Cracov.

The study included a dental check-up with a dental diagram (Appendix A), and supplementary medical documentation (consent to conduct the study and personal data processing, questionnaires). Data on height, weight, age, gender, as well as eating habits and dietary supplements were completed by the parent or legal guardian of the child during the visit to the dentist.

The exclusion criteria were: complex comorbidities (caries complications: inflammation in the oral cavity, abscesses, pulp inflammation, *C. albicans* infections (thrush), injuries of the oral mucosa), intellectual disability, undergoing orthodontic treatment, lack of informed consent from their legal guardians, coexistence of diseases that may affect the assessment of taste (diagnosed hyposmia/anosmia, nutritional neophobia, type 1 diabetes, infectious diseases, especially those requiring antibiotic therapy, condition after antibiotic therapy, infectious or allergic rhinitis, symptoms of Oral Allergic Syndrome (OAS), anaphylaxis or other allergic reactions, neurological diseases, mechanical damage to the central or peripheral nervous system, especially cranial nerves, neoplastic diseases, with particular emphasis on cancers of the oral cavity and tumors of the central nervous system, oncological treatment, head injuries, craniofacial injuries), lack of cooperation between the child and the physician, impaired salivary flow, and voluntary withdrawal of the participant or its legal guardian at any stage of the research. A fresh (unhealed) wound after tooth extraction or other dental intervention was also a disqualifying factor.

The study was conducted in accordance with international standards in clinical trials and the Helsinki Declaration. Informed written consent was obtained from all participants in the study/legal guardians of minor participants after obtaining approval from the Bioethics Committee of the Jagiellonian University in Cracov (No. 1072.6120.174.2019).

### 2.2. Data Collection

The collection of clinical data was performed by experienced dentists after training by a principal investigator specializing in pediatric dentistry with research experience. Caries assessment and other elements of the study, such as the assessment of sweet taste perception and the collection of questionnaire data, were carried out in successive stages; the first was the meeting of the principal investigator with the rest of the dentists to determine the individual steps of the study, and the second meeting was where data collection from patients was simulated. The validation of the taste test protocol in clinical conditions was carried out on previously prepared sucrose solutions by all examiners with the participation of volunteers. Regarding the clinical evaluation of caries, standardized examination protocols and questionnaire forms were used.

During the first visit, trained dentists collected demographic data and medical information using a simple questionnaire (Appendix A). The assessment of the sweet taste perception and the clinical assessment of the oral cavity were performed during the first medical visit, while oral microflora was assessed with the material being collected during the next visit. Data from each participant in the study were blinded and coded. Collected clinical and questionnaire data sets were kept separately until the end of the study. After completion of all analyses, a complete statistical analysis and interpretation of the obtained test results was performed.

### 2.3. General Data about Children

Data on height (cm) and body mass (kg) were obtained on the basis of the information included in the questionnaire completed by the parents (Appendix A). Body mass index (BMI) was then calculated based on the formula:

(1) $$BMI=\;\frac{body\;mass\;\left(kg\right)}{{\left(height\;\left(m\right)\right)}^{2}}$$

Consequently, participants were classified into groups of under-, normal and overweight children based on BMI percentile grids for the population of Polish children [70].

### 2.4. Analysis of Eating Behavior

The study of the eating habits of children was conducted using a proprietary questionnaire addressed to the parents or legal guardians [71,72]. The survey consisted of nine questions. Its purpose was to obtain information on the eating behavior that may be particularly conducive to the development of dental caries, such as consumption of sweets or drinks other than tap water. The questions also concerned the regularity of meals, type of diet (traditional or vegetarian), as well as snacking between meals.

### 2.5. Assessment of Sweet and Bitter Taste

The perception of sweet taste was assessed using a specific taste sensitivity testing method, based on tasting distilled water and diluted solutions of sucrose (sweet taste) and caffeine (bitter taste) without swallowing. Sucrose of analytical quality (Chempur, Poland) and sterile distilled water were used to prepare the solutions (under sterile conditions), which were then stored in the refrigerator until the day of testing. These actions were aimed at preventing the growth of microorganisms that could alter the taste of the solutions. The solutions were warmed to room temperature prior to testing.

The participants of the study (and their legal guardians) were not informed what substances were dissolved in the samples, the concentrations of the prepared samples, and the order of the solutions and the coding of the samples. This procedure was intended to avoid the suggestion of a taste score. The concentrations of sucrose and caffeine solutions were selected according to International Organization for Standardization (ISO) 3972:2011 [73] and are presented in Table 1.

The level of taste preferences and the threshold rating for a given flavor type were combined and evaluated simultaneously. The proprietary method had previously been calibrated in pilot studies (unpublished research by Jurczak et al., 2019). The variables identified in this test are the concentration threshold for sweet taste (the point at which the child is able to identify the presence of sucrose in a solution and determine its presence in relation to water) and taste preferences (children were asked if they sensed a taste, if the solution tasted the same as water, whether the taste was pleasant or not, and were shown appropriate graphic signs, i.e., smiling or sad faces).

Before the scheduled visit, participants were asked not to eat, drink, or brush their teeth at least one hour before the examination. Subsequently, the children were offered eight sucrose solutions in the concentration range from 0.34 g/L (1.0 mM/L) to 12 g/L (35.1 mM/L). The child was given 10 mL of each sucrose solution in disposable plastic cups in increasing order of concentration. Subsequently, the child tasted the solutions for at least 5–10 s in order to stimulate all the taste buds, after which the solution was spat out. Children were asked to define both the threshold of perceived sucrose concentration and the perceived taste preference. Children rinsed their mouths with distilled water between each concentration of the tested formula. If the child was unsure of their feelings, the test was repeated after 10 min had elapsed from the first examination.

For statistical analysis of the obtained data, children were divided into groups based on the reported level of sweet taste perception. Depending on the concentration of the selected threshold and the taste preferences, there were two groups, i.e., children who preferred the solution in the range up to 4 g/L, and the second group, when the preferred sucrose solution concentration was >4 g/L.

The study was carried out during a visit to the dentist’s office, where neutral conditions conducive to focusing on taste stimuli were not ensured. This constitutes a limitation of the study.

### 2.6. Clinical Evaluation of Caries

The dentition condition was assessed according to WHO recommendations and criteria [74]. The clinical examination was carried out using a diagnostic mirror and a WHO probe under artificial lighting (dental unit lamp).

Caries incidence was determined using the dmft index, which is the sum of the number of permanent/deciduous teeth with current carious lesions (d), extracted (m), and filled (f) due to caries, while the severity of caries was assessed based on the International Caries Detection and Assessment System (ICDAS II) [75].

In the ICDAS assessment, the dentists conducting the study were trained by the principal investigator; the results were calibrated and data recorded. The degree of advancement of carious changes in the teeth of the examined children was assessed after cleaning and drying. Code 0 corresponds to no carious lesions; codes 1 and 2: early carious lesions limited to enamel in the form of opacity; lesions limited to enamel with loss of its continuity, without dentine exposure—code 3; while carious lesions including enamel and dentin with/without tissue defect—codes 4–6 according to the ICDAS II classification (codes 5 and 6 indicate the presence of a cavity with discernible dentin).

Carious lesions recorded in the ICDAS II system were divided according to the severity of the level of surface lesions ICDAS II 1,2,3 (initial caries affecting the enamel without visible dentin) and ICDAS II 4,5,6 (caries affecting dentin). Mean scores for each patient were calculated relative to the ICDAS II scores for all teeth. In the pilot studies, Cohen’s kappa coefficients were determined for the researchers conducting the ICDAS II assessment, obtaining a mean for the reproducibility of the test results at the level of k = 0.79–0.86.

### 2.7. Collection of Samples and Microbiological Analysis

Children were pre-administered with sterile saline to rinse their mouth. The obtained washings in a sterile container were sent to the laboratory for microbiological analysis before the actual taste test.

Microbiological analysis was based on quantitative and qualitative assessments of aerobic and micro-aerophilic microbiota of the children’s mouth. For this purpose, the method of inoculating serial dilutions on solid media, both selective and non-selective, was used.

At the beginning of the follow-up visit to the dentist, each child was given 10 mL of sterile distilled water to rinse their mouth.

The oral cavity is a complex ecosystem with numerous ecological niches, and planktonic mycobiota suspended in saliva is one of these. Its microbiological composition is influenced by the composition of the microbiome of other niches, including the surface of the teeth, gums, and mucous membranes of the cheeks [41,76].

The choice of mouth rinse was determined because of the importance of the oral planktonic microbiome [77] in shaping both the surface microbiome of the tongue and the taste sensation. In addition, the authors wanted to obtain a relatively homogeneous suspension of microorganisms belonging to different oral habitants, i.e., microbes forming dental biofilms (mainly differentiated microflora, both microaerophilic and absolutely anaerobic microorganisms) [78] associated with the development of caries in children, salivary microorganisms [79,80,81] which are considered an indicator of the entire microbiome of the oral cavity and thus may reflect dysbiosis in dental plaque biofilms initiating clinical symptoms of ECC [82], and microorganisms of the tongue (differentiated microflora, relatively and absolutely anaerobic) connected with taste buds and the perceived sweet taste [83].

An additional support for this approach is the high species diversity, taking into account the majority of oral habitants responsible for both the development of caries and taste sensations. This approach is also supported by the relatively similar number of microbial species obtained as opposed to the selected location, which would not allow for the reproducibility and homogeneity of the microbiological composition of the tested materials. In addition, the authors wanted to obtain a stable (resident) microflora remaining in a state of dynamic equilibrium with the host, which would be impossible if the study was limited to only one location, e.g., the surface of the tongue, where the taste buds are located.

Moreover, discussion of the caries process cannot be focused solely on the microflora of the tongue surface, as the species found there determine the development of caries to a lesser extent due to the mainly anaerobic environment. Similarly, the number of pathogenic species occupying specific ecological niches is not synonymous with the development of caries in the oral cavity.

No other work has been found in the taste testing literature that uses a similar methodology. The use of mouth rinses instead of saliva is certainly a limitation of this study. On the other hand, the use of this method enables the isolation of species from more niches.

Rinsing took approximately 1 min. After this time, the washings were spat out into a sterile container. The samples were sequentially shaken by gentle vortexing and then sonicated for 30 s. Then, serial dilutions of the stock solution were prepared in sterile physiological saline. Samples from the obtained dilutions were inoculated on 5% blood agar plates (Columbia Agar and Schaerdler Agar, Graso Biotech, Poland) and subsequently on selective media used to detect more demanding groups of microorganisms. After selection, the following media were used: chromogenic CHROMagar™ agar supporting the growth of Candida and yeast, Enterococci-supporting agar (BD Enterococcosel Agar), BD MacConkey II Agar selective medium for coli bacteria (CCA), chromogenic agar for the isolation and differentiation of Enterobacteriaceae, and HLR-S medium for *S. mutans* isolation. Inoculated media were incubated under micro-aerophilic conditions in the presence of 5% CO_2_ at 37 °C for 24–48 h. Based on their morphology, yeast colonies, Enterococci, Enterobacteriaceae, Streptococci, and Staphylococci were grown on selective media, where they were counted. Pure isolates were placed in growth and differentiation media for further identification. Initial identification was performed on the basis of microscopic examination of Gram-stained slides and various biochemical identification results (API System Candida; API STREP for Streptococci and API Staph by bioMérieux, France for staphylococci). Based on the color and morphology of the yeast colonies on CHROMagar^TM^ Candida, isolates were identified as *Candida albicans*.

Bacterial and fungal species obtained during the cultivation were identified by means of MS MALDI TOF mass spectrometry (Bruker Daltonik, Germany), in accordance with methods described earlier [84,85].

### 2.8. Statistical Analysis

Perception of sweet taste (both sweet taste threshold and taste preferences) and assessment of caries prevalence (DMFT/dmft, initial caries and caries manifestation) were analyzed using IBM^®^ SPSS^®^ (IBM, Chicago, IL, USA, version 26.0). The mean, standard deviation, range, and frequency of the variables were calculated for each group separately. The compliance of the distribution of the analyzed variables with the normal distribution was checked using the Shapiro–Wilk test. Student’s *t*-test, Mann–Whitney U, or chi-square test were used to evaluate the differences in the studied variables between the groups. The odds ratio and the 95% confidence interval were calculated using logistic regression. A *p* value of <0.05 was considered statistically significant.

## 3. Results

### 3.1. Study Group

There were no significant differences between the sexes in terms of the incidence of caries (*p* = 0.701; chi-square test). Further evaluation was carried out between the study groups without reference to sex.

The mean age of the respondents was 4.76 ± 1.18 years (mean ± standard deviation (SD)); while in the group of children with caries it was 4.73 ± 1.09 years and 4.79 ± 1.28 years without caries. These values also did not differ significantly (*p* = 0.8315; Mann–Whitney U test).

### 3.2. Body Weight Results

Mean BMI of all study participants in was 15.23 ± 2.39 kg/m^2^ (Table 2). Body height and weight did not differ in the studied groups, as is shown in Table 2. Additionally, in Table 3, the BMI assessment was presented with the division into the adopted scales of the BMI in the studied groups. Study participants were divided according to the adopted scales for assessing children in the study groups, i.e., underweight, normal body weight, overweight, and obesity. According to this, 52.4% of the study participants had a normal body weight and about 16% showed an increased body weight (Table 3). These differences, however, were not sufficiently statistically significant so as to perform classification of the examined parameters based on the adopted scales.

### 3.3. Assessment of Eating Behavior

Among a number of questionnaire questions, attention was drawn to the existence of statistically significant differences in terms of snacking between meals and the frequency of eating sweets in the group of children with caries compared to controls (*p* < 0.001; chi-square test). In children eating between meals, a lower sensitivity (higher threshold concentration) to the sensation of sweet taste, i.e., sucrose (4.52 ± 2.64 g/L), was observed compared to children who did not eat between meals (3.38 ± 2.62 g/L; *p* = 0.0273, U Mann–Whitney test). On the other hand, the highest percentage of children eating sweets (once a day to several times a day) was in the group of children with caries 96.9%. In the group of children without dental caries, the largest percentage was constituted by children who did not eat sweets at all or not every day (83.9%; Table 4).

The odds ratio for the risk of dental caries was calculated by grouping the children into two groups: those who did not eat sweets and those who ate sweets once a day or several times a day. Children who ate sweets once a day or several times a day were more than 160-times more likely to develop caries compared to the children who did not eat sweets or ate sweets daily (OR = 161.2, 95% CI 16.9–1536.5).

### 3.4. Evaluation of Sweet Taste

**Figure 1:**
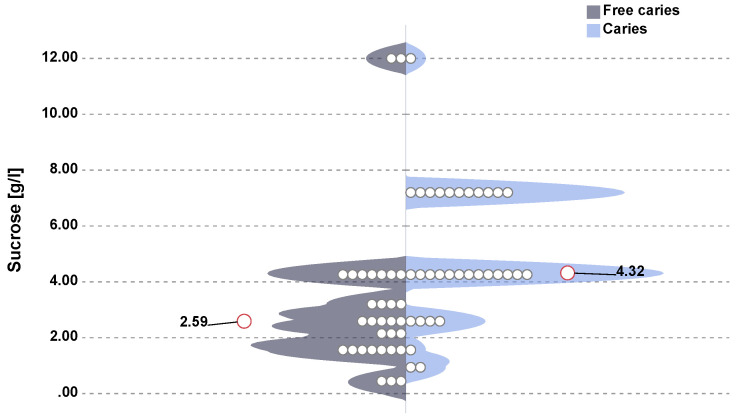
Sucrose sensation threshold (individual observations and median sucrose concentrations divided into groups). In children with caries, a statistically significant higher sucrose sensation threshold was found in relation to children without caries (median (lower quartile–upper quartile)) (4.32 (4.26–7.20) vs. 2.59 (1.56–4.32)), *p* < 0.001, U Mann–Whitney test.

The assessment of sweet taste perception in children showed the highest mean value of this index among children with caries. Twenty-three children with caries were sensitive to sucrose concentrations in the range 4.32–7.20 g/L, while caries-free children were sensitive to sucrose in concentrations 1.56–3.20 g/L. The highest preferable concentration of sucrose for all children was 4.32 g/L, similar to that in the group of children with caries. In the group of children without caries it was 1.56 g/L. When assessing the sensitivity thresholds to sweet taste (sucrose sensation threshold) in both groups, a statistically significant difference was observed for the concentration of 4.32 g/L (*p* = 0.0015, chi-square test) in children with caries and for the concentration 2.59 g/L (*p* = 0.0005, U Mann–Whitney test) in controls (Figure 1).

### 3.5. Clinical Examination

Caries assessment results are presented as dmft and ICDAS II scales. Children with caries had significantly higher dmft (7.97 ± 4.08) compared to children without caries (0 ± 0; *p* < 0.001; U Mann–Whitney test). Similarly, in the case of the ICDAS II assessment scale, children with caries had an index value at the ICDAS II level of 5.03 ± 1.36.

### 3.6. Microbiology

**Figure 2:**
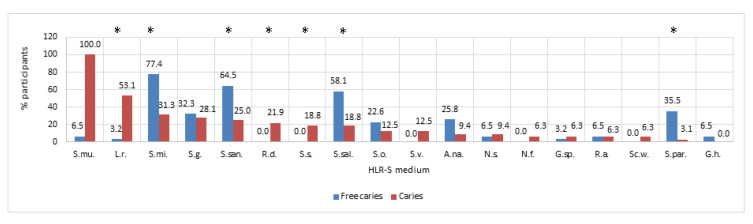
Prevalence of particular species of bacteria isolated from mouth rinses from children with caries (Caries) and controls (Free Caries) on a modified selective Ritz HLR-S medium (consisting of Trypticase soy agar, sucrose, bacitracin, polymyxin B sulfate, and crystal violet) [13]. S.mu.—*Streptococcus mutans*, L.r.—*Lactobacillus rhamnosus*, S.g.—*Streptococcus gordonii*, S.san.—*Streptococcus sanguinis*, R.d.—*Rothia dentocariosa,* S.s.—*Streptococcus sorbinus,* S.sal.—*Streptococcus salivarius*, S.v.—*Streptococcus vestibularis*, A. na.—*Actinomyces naeslundi*, N.s.—*Neisseria subflava,* N.f.—*Neisseria flavescens,* G.sp.—*Gemella sp.*, R.a.—*Rothia aeria*, *Sc.w.*—*Scardovia wiggsiae,* S.par.—*Streptococcus parasanguinis*, G.h.—*Gemella haemolysans*, * *p* < 0.05.

**Figure 3:**
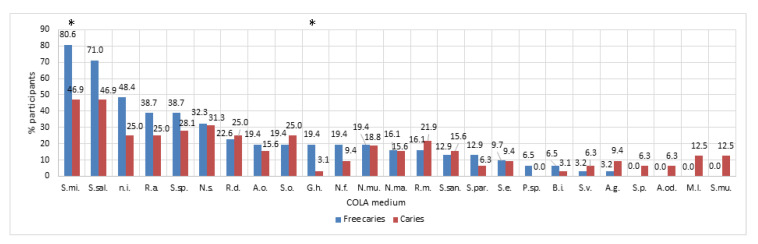
Results of the qualitative assessment of the composition of the oral microbiota of the studied children via inoculation of serial dilutions on the nutrient Columbia Agar medium with 5% sheep blood. Percentage of the occurrence of particular species of bacteria isolated from mouth rinses from children with caries (Caries) and controls (Free Caries). S.mi.—*Streptococcus mitis,* S.sal.—*Streptococcus salivarius,* n.i.—*not identified,* S.sp.—*Streptococcus sp.*, N.s.—*Neisseria subflava*, R.d.—*Rothia dentocariosa*, A.o.—*Actinomyces oris,* S.o.—*Streptococcus oralis*, G.h.—*Gemella haemolysans*, N.f.—*Neisseria flavescens*, N.mu.—*Neisseria mucosa*, N.ma.—*Neisseria macacae*, R.m.—*Rothia mucilaginosa*, S.san.—*Streptococcus sanguinis*, S. par.—*Streptococcus parasanguinis*, S.e.—*Streptococcus epidermidis*, p.sp.—*Prevotella* sp., B.i.—*Bacillus indicus*, S.v.—*Streptococcus vestibularis,* A.g.—*Actinomyces graevenitzii,* S.p.—*Streptococcus pyogenes*, A.od.—*Actinomyces odontolyticus*, M.l.—*Micrococcus luteus,* S.mu.—*Streptococcus mutans*, * *p* < 0.05.

The results of the microbiological analysis showed the presence of specific microbiological profiles in the studied groups, as is shown in Figure 2 and Figure 3.

In the case of caries-free children, the percentage of the most common species of bacteria included the following: *Streptococcus mitis* (S.mi.; 80.6%); *Streptococcus salivarius* (S.sal.; 71%); *Neisseria subflava* (N.s.; 32.3%); *Rothia dentocariosa* (Rd; 22.6%); and others presented in Figure 2. In the case of the study group, the share of the following additional species was noted: *Streptococcus* sp. (S.sp.; 28.1%); *Rothia mucilaginosa*; *Streptococcus sanguinis*, etc. (Figure 2).

In the case of *S. mutans* isolated on a selective medium from the washing samples of children with and without caries, there was a statistically significant difference in the level of logCFU/mL (*p* < 0.001; U Mann–Whitney test). In children with caries, the number of isolated *S. mutans* was 5.57 ± 0.74 logCFU/mL, while in children without caries the number was less than 0.16 ± 0.89 logCFU/mL of *S. mutans*.

In the *S. mutans*-positive samples, the mean percentage was 100% in the caries group and 6.45% in the controls. *S. mutans* was identified in all 32 children with ECC, but in only 1 of 31 caries-free children.

Among other isolated species of bacteria, significant differences between children with and without caries were noted for *Lactobaillus rhamnosus* (53.13% vs. 3.23%). In the case of fungi isolated on the differentiating medium, attention was drawn to the existence of a significant difference between *C. albicans*, which was more often isolated in children with caries compared to children without caries (*p* = 0.0166; U Mann–Whitney test).

### 3.7. Odds Ratio

In order to assess the effect of the taste of sucrose on the occurrence of caries, excess body weight, the presence of *S. mutans*, or frequent consumption of sweets, the odds ratio and the 95% confidence interval were calculated. Children with an elevated sucrose threshold were almost 9-times more likely to develop caries (OR = 8.73; 95% CI 2.72–27.99), and more than 10-times more likely to develop *S. mutans* presence (OR = 10.21; 95% CI 3.11–33.44), as well as an almost 9-fold higher chance of consuming sweets more often (OR = 8.57; 95% CI 2.67–27.56). There was no significant correlation between the elevated sucrose threshold and the occurrence of excess body weight in the studied group of children (OR = 0.51; 95% CI 0.12–2.08) (Table 5).

## 4. Discussion

Among many factors influencing the increased risk of caries, genetic susceptibility plays an important role, increasing the appearance of the problem by about 50% [86,87,88]. Having a genetic background predisposition to prefer a particular taste may be related to the preference or rejection of certain foods by children. In particular, genes (*gnat3*, *slc2a4*, *tas1r1*, and *tas1r2*) related to the sweet taste receptor, glucose transport (GLUT2), or specific clinical genotypes, have a decisive influence on the development of specific preferences regarding taste [88]. Systematic reviews show that, despite educational programs or interventions concerning nutritional behavior among children, the problem still exists [89,90,91,92]. It turns out that taste preferences regarding the willingness to take sugar determine the increased risk of dental caries and overall poor health much more than promoting a healthy lifestyle by limiting sugar consumption, which seems to be less decisive for the final success in fighting the disease.

It seems obvious that consuming excessive amounts of sugars contained in sweetened drinks, falling asleep with a bottle full of liquid containing carbohydrates (milk, juice), feeding a bottle before falling asleep or often during the day, prolonged night breastfeeding, or prolonged periods of contact with the bottle induces poor nutritional behavior later on, increasing the risk of caries in preschool children [93]. Children’s consumption of sugar of over three daily servings has also been associated with an increased risk of early childhood caries [94].

The conducted study showed the existence of a strong positive correlation between the perception of a sweet taste and the occurrence and severity of caries in preschool children.

Out of 63 children examined at the Department of Pediatric Dentistry, University Dental Clinic in Cracov, 46% of children had a sweet taste threshold of <4 g/mL and 54% of children >4 g/mL. Similar results for school-age children and adolescents have been obtained by other researchers [95,96,97]. Low sucrose sensitivity correlated with higher mean dmft. The conducted research has shown that the amount of consumed sugar influences the development of the threshold of this taste in children. The more sugar is consumed, the higher is the sensitivity to the sweet taste, and this in turn can lead to even higher sugar consumption, thereby increasing the risk of caries [95]. Similar observations were made by Robino et al., where they indicated the existence of a similar correlation between the threshold of the perception of sweet taste and dmft [58]. In a study by Furquim et al. the authors did not observe a significant difference in the perception of a sweet taste between the groups of 12-year-old children with low and high severity of caries [96]. Similarly, no significant correlations were found in the studies by Maciel et al. and Bretz et al. [79,98].

The greater incidence of tooth decay in children with low sensitivity to sweet taste may result from the more frequent consumption of sweet food and drinks. As a result of a long-term, regular consumption of sweet foods, the receptors adapt to these stimuli. Reducing the sensitivity to sweet taste means that a higher dose of sugar is needed to obtain a satisfactory level of sweetness [95,99].

Therefore, the dietary determinants of caries, resulting from the proven strong relationship of excessive, mainly in terms of frequency but also quantity, sugar supply and the risk of its occurrence, seem to be crucial [24]. The authors of this study drew attention to the significantly more frequent consumption of sweets in the group of children with caries compared to those without caries, among whom over 83% did not eat sweets every day. Therefore, the obtained results clearly indicate another significant relationship in this respect between the perception of the sweet taste and the frequency of consuming sweets. Children with higher sucrose sensitivity thresholds and its higher preferred sucrose concentrations consumed sweets significantly more often than children with lower sensitivity thresholds, more sensitive ones, preferring lower glucose concentrations. This is in line with the evidence available in the literature on the effect of individual differences in the perception of sweet taste on eating habits. An individual’s ability to sense sweetness in the mouth is believed to be one of the factors influencing food acceptance, and therefore taste may play an important role in modulating it and hence, in food selection [100]. It has been found that choices concerning the type of food are made mainly on the basis of taste [101,102]. Evidence suggests that excessive consumption of sucrose is more detrimental to metabolic changes by increasing the craving for sweetness and contributing to obesity and sugar addiction via neuro-adaptation [103].

Another explanation for the observed relationship between the intensity of the sweet taste and its preference is fluctuating glucose concentration in the body. People who perceive glucose solutions as sweeter consume less carbohydrates, eat fewer sweet meals, and have less liking for sugary drinks compared to people who perceive glucose solutions as less sweet. This confirms that people who like the sweetness of high glucose levels have a higher habitual intake of energy and sugars [104]. Individual differences in sweetness and sweet taste responses may therefore be an indicator of differences in preferred diet and likely food choice patterns [104].

In the context of the obtained results, it seems interesting to verify the hypothesis as to whether the perception of sweet taste may be modulated by reduced sugar consumption. There is evidence available in the literature to show that changes in the consumption of simple sugars affect the perceived intensity of sweet taste [105]. Another issue is how the observed changes in the perception of sweetness intensity influence eating behavior, i.e., whether reduced sugar consumption ultimately changes preferences for sweet foods [106]. In addition, there have been reports challenging the role of perceived sweetness intensity in eating behavior related to sugar-containing foods in adults [106].

The aim of a systematic review of available studies assessing the relationship between sweet taste function or perception and dietary habits by Tan et al. was an attempt to determine which type of taste (sweet, bitter, sour) is most closely related to food intake in healthy adults [107]. It turned out that the results of the sweet taste sensitivity and intensity study showed a slight correlation with the consumption of carbohydrates, while the obtained values of the preferred sugar concentrations were much more likely to correlate with the consumption of sweet foods, but did not give consistent results [107]. Only a small fraction of the available studies showed a significant negative association between sweet taste scores and diet, taste sensitivity and intensity, and sugar consumption, while preferred concentrations were positively associated with sugar intake in all studies except one. It seems that the measurement of taste preferences provides a relatively better insight into eating behavior compared to the assessment of sensitivity to a given taste and its intensity [107].

In this study, the perception of sweet taste correlated, apart from caries, with the amount of *S. mutans* isolated from the mouth washes. The reason for such a result may be both the consumption of more sweets and the ongoing caries process in children with low sensitivity to a sweet taste. Foods rich in sucrose have a positive effect on the development and formation of biofilm by *S. mutans* [26,108]. Increasing the sucrose concentration in the solution above 1% significantly increased the adherence of *S. mutans* cells to the surface. This trend concerned strains isolated from children from both ECC and control groups [109]. With an increase in sucrose concentration, the production of extracellular polysaccharides (EPS) and lactic acid by *S. mutans* also increases [110]. This, in turn, initiates the production of glucans by *S. mutans* which are critical to promoting biofilm formation and caries associated with the presence of *C. albicans* [111].

Children who were less sensitive to lower sucrose levels showed an increased presence of cariogenic *S. mutans*. Moreover, it was found that the intensity of caries (dmft) was higher in children showing a higher sweet taste threshold (>4 g/mL) of 73.5% and an increased number of *S. mutans* streptococci (76.5%). Additionally, frequent consumption of sweets between meals increased the incidence of caries by 161.2-times in relation to children who did not eat sweets between meals. The incidence of *S. mutans* also showed a strong two-way correlation with the incidence of dental caries. The frequency of consuming sweets was closely related to caries and the threshold of perceiving sweet taste. It has been known for a long time that the composition of the gastrointestinal microbiota, including oral cavity and intestines, is influenced by persistent eating habits, which explains the existence of structural differences in the composition of the total number of microbial species inhabiting it [112]. Interactions between a diet rich in simple sugars and the presence of cariogenic species of microorganisms, including *S. mutans*, lactobacilli, and *C. albicans*, may contribute to bacterial dysbiosis (altered microbial diversity) with a predominance of acid-producing and acid-tolerant microorganisms. As a consequence of dysbiosis of the oral cavity microbiome, there are disturbances in the amount of bacterial metabolites (in the context of the activity of microorganisms that constitute dental biofilm) in saliva or plaque, determining the subsequent host inflammatory reaction and further development of caries [36,113]. A classic example of the influence of metabolites of bacterial origin are the interactions of these factors with extracellular TLR receptors, which mediate microbial-host interactions through signaling pathways (NF-κB and mitogen-activated protein kinases (MAPK)), affecting oral tolerance to the benefit of the host or promoting systemic inflammation [114].

The results obtained in this study indicate the presence of specific bacterial profiles and the prevalence of cariogenic species such as *S. mutans* or *C. albicans* in children with lower sensitivity to sweet taste, who develop caries. This study appears to be the first to attempt to determine the relationship between cariogenic species and the sweet taste preference. Among the studies existing in the literature, a number indicate a clear relationship between caries and *S. mutans* bacteria, poor oral hygiene, and difficult access to dental care [115]. Based on the above-mentioned, the results of the caries-free children obtained in this study generally show lower or no detection of *Streptococcus mutans*, which is consistent with our observations. In addition, this study confirmed the role of *Streptococcus mitis* as a factor supporting the development of oral biofilms, with its dominant share in the group of caries-free children, in contrast to the work of Johansson et al. [116], where it was not detectable in caries-free children. These differences may result from the microbiological methods applied. *Streptococcus mitis* is a commensal bacterium inhabiting the oral cavity and upper respiratory tract of humans [11,12], which seems to colonize infants in the first days of life [13] in proportions that show high individual variability [14].

Pioneering colonization by *S. mitis* strains may also initiate an adaptive cross-response to PcsB expressed by later colonizers. The current findings on PscB of *S. mitis* raise further interest in defining the effects of an immune response to PcsB proteins on oral ecology and host microbiota homeostasis [117].

In addition, the potential for low pH probably differs significantly between oral streptococci species and is least likely among *S. mitis* strains. This sheds new light on the revision of the concept and composition of “low pH streptococci” [118].

This feature also shows synergy with a sweet taste preference and reduced sucrose sensitivity threshold in children suffering from caries.

Among the mechanisms of sweet taste signaling identified in the oral cavity, there are also those operating in the gastrointestinal system. These can influence the feeling of fullness or the development of overweight and obesity at a later age [119]. The relationship between BMI and the perception of sweet taste has been discussed in a few studies [97,120,121]. It has been shown that in European children, the sweet taste preference correlates with BMI values [121]. In other studies, just as in ours, no such relationship has been found; there is also no correlation between the BMI value and dental caries [97,120]. However, a study by Quadri et al. showed a correlation between BMI and the occurrence of dental caries in school-age children [122]. The correlation was clear, especially for children from families with high socioeconomic status, which could have resulted from the higher consumption of sweets in this group.

The assessment of the composition of the oral microflora using classical microbiology techniques is one of the limitations of this study. The current research focuses on the use of omics techniques and 16S rRNA sequencing analysis, which allow much better monitoring of microorganisms found in individual ecological niches of the oral cavity. These methods can confirm and extend understanding of the microorganisms involved in vertical and horizontal transmission in the development of dental caries in larger populations. In addition, these techniques enable the discovery of new species, especially when the identification of the strain is possible only through gene sequence analysis. Thanks to sequencing techniques, it has become possible to identify anaerobic microorganisms associated with caries or related species such as *Scardovia wiggsiae* of the Actinomyces/Bifidobacterium family and Actinomyces species, which are important in cariogenic acid production and tolerance.

This is of great importance because the actual diversity of the oral microbiome cannot be fully disclosed by culture-based methods [123], which is especially important in the study of diseases related to microbiota dysbiosis.

Another limitation of this study is the small group size, especially for the odds ratio. Nevertheless, this research will be continued.

## 5. Conclusions

Current understanding of the prevention and treatment of social diseases such as dental caries should take into account analysis of the correlation of many variables or information affecting oral health. The accuracy of the developed models for caries risk assessment, combined with taste preferences, should be the basic element in predicting susceptibility to this disease.

Knowledge and observations of taste preferences from the first days of a child’s life may lead to the development of a phenotype of the microbiological nature of caries, which together could allow the use of this information to prevent caries in the youngest groups from the first months of a child’s life.

The perception of a sweet taste has an impact on eating habits, especially the frequency of consumption of foods rich in sucrose, and therefore significantly affects predisposition to the development of caries.

Current results of the conducted study suggest the possibility of using preference assessment to perceive the sweet taste concentration in children as a diagnostic tool for the early detection of increased susceptibility to caries through microbial dysbiosis towards specific species of microorganisms.

## Figures and Tables

**Table 1:** Series of concentrations of sucrose solutions used in the sensory test. Based on ISO 3972:2011 [73].

Sucrose (g/L)
0.34
0.55
0.94
1.56
2.59
4.32
7.20
12.00

**Table 2:** Analysis of differences between age, weight, height, and BMI in the studied groups.

Total *N* = 63.				Controls *n* = 31			With Caries *n* = 32			
	X	SD	Me	X	SD	Me	X	SD	Me	*p*
Age	4.76	1.18	5.00	4.79	1.28	5.00	4.73	1.09	5.00	0.831 ^A^
Body mass (kg)	18.84	4.58	18.00	19.47	5.39	19.00	18.23	3.62	17.75	0.491 ^B^
Height (cm)	111.11	12.16	110.00	110.10	13.67	109.00	112.09	10.63	110.00	0.519 ^A^

BMI—body mass index, X—mean, SD—standard deviation, Me—median, ^A^—Student *t*-test result, ^B^—U Mann–Whitney test result. *p* < 0.05 is statistically significant. Mean ± SD are presented; different letters in superscript indicate significant differences between the groups.

**Table 3:** The prevalence of the BMI index in the adopted scales for assessing children in the studied groups.

	Total			Controls			With Caries			Chi-Square Test
BMI	*n*	%	Cumulative %	*n*	%	Cumulative %	*n*	%	Cumulative %	
Underweight	20	31.7	31.7	8	25.8	25.8	12	37.5	37.5	
Normal weight	33	52.4	84.1	15	48.4	74.2	18	56.3	93.8	0.0575
Overweight	9	14.3	98.4	8	25.8	74.2	1	3.1	96.9	
Obesity	1	1.6	100.0	0	0.00	100.0	1	3.1	100.0	
Total	63	100.0		31	100.0		32	100.0		

The differences were not statistically significant (*p* = 0.057, chi-square test). BMI—body mass index

**Table 4:** Frequency of eating sweets in the studied groups of children.

	Total			Controls			With Caries			Chi-Square Test
Sweets intake	*n*	%	Cumulative %	*n*	%	Cumulative %	*n*	%	Cumulative %	
No	9	14.3	14.3	9	29.0	29.0	0	0	0.0	
Daily	18	28.6	42.9	17	54.8	83.9	1	3.1	3.1	<0.0001
Once a day	21	33.3	76.2	3	9.7	93.5	18	56.3	59.4	
Several times a day	15	23.8	100.0	2	6.5	100.0	13	40.6	100.0	
Total	63	100.0		31	100.0		32	100.0		

The differences were statistically significant (*p* < 0.001, chi-square test).

**Table 5:** Factors associated with sweet taste perception.

Variables	Category	%	
		Sucrose	Sucrose
		<4 mg/L	>4 mg/L
Caries	no	75.9	26.5
	yes	24.1	73.5
	OR	1	8.73
	95% CI		2.72–27.99
BMI (overweight or obesity)	no	79.3	88.2
	yes	20.7	11.8
	OR	1	0.51
	95% CI		0.12-2.08
*S. mutans* presence	no	75.9	23.5
	yes	24.1	76.5
	OR	1	10.21
	95% CI		3.11–33.44
Consumption of sweets	no	69	20.6
(once a day or several times a day)	yes	31	79.4
	OR	1	8.57
	95% CI		2.67–27.56

## Appendix A

1. What meals and at what times during the day does your child eat? Please enter in the table below the time when the child normally eats a given meal. If the meal is not consumed, please leave the field blank.
  **Meal**	Breakfast	Lunch	Dinner	Tea-time	Supper	II supper/snack
  **Time**						
2. Does your child eat between meals?
  □ Yes
  □ No
3. How often does your child eat sweets (candies, chocolates, cookies, sweetened milk products) during the day?
  □ Not at all
  □ Sweets do not appear every day
  □ Once a day
  □ Several times a day
4. Does your child eat in the evening or at night after brushing their teeth?
  □ Yes
  □ No
5. Does your child consume any beverages other than plain water during the day?
  □ Yes
  □ No
6. Does your child consume any beverages other than plain water in the evening or at night after brushing their teeth?
  □ Yes
  □ No
7. Is your child on a vegetarian/vegan diet?
  □ Yes
  □ No
8. Does the child receive any dietary supplements?
  □ Yes
  □ No

If yes, please provide the brands of the supplements used and, if possible, the dose:

9. Please complete the anthropometric parameters of your child:
  **Age:**		**Sex:**	
  Body mass:		Height:	
